# Immature Platelet Fraction as a Sensitive Biomarker in Neonatal Sepsis: Diagnostic Performance Preceding Thrombocytopenia

**DOI:** 10.3390/children12070931

**Published:** 2025-07-15

**Authors:** Ilkay Er, Medeni Arpa

**Affiliations:** 1 Department of Pediatrics, Division of Neonatology, Faculty of Medicine, Recep Tayyip Erdogan University, Rize 53020, Turkey; 2 Department of Medical Biochemistry, Faculty of Medicine, Recep Tayyip Erdogan University, Rize 53020, Turkey; medeni.arpa@erdogan.edu.tr

**Keywords:** neonatal sepsis, immature platelet fraction, promising hematologic biomarker, diagnostic performance

## Abstract

**Background**: Early and accurate diagnosis of neonatal sepsis remains a clinical challenge due to nonspecific signs and limitations of conventional biomarkers. The immature platelet fraction (IPF), a novel hematologic parameter reflecting thrombopoietic activity, has emerged as a potential early sepsis indicator. This study aimed to evaluate the diagnostic value of IPF in neonatal sepsis prior to the onset of thrombocytopenia. **Methods**: This prospective study enrolled neonates with early-onset sepsis (EOS), late-onset sepsis (LOS), and healthy controls. IPF, C-reactive protein (CRP), procalcitonin (PCT), and hematologic indices were measured at diagnosis and 48–72 h post-treatment. Diagnostic performance was evaluated via ROC curve analysis, and correlations between IPF and inflammatory/hematologic markers were examined. IPF levels were also compared based on blood culture results. **Results**: IPF levels were significantly higher in both EOS (n: 56) and LOS (n: 50) groups compared to controls (n: 44) (*p* < 0.001). ROC analysis showed excellent diagnostic performance, with AUCs of 0.98 (EOS) and 0.99 (LOS). Following antibiotic treatment, IPF levels declined significantly (*p* < 0.001), supporting its dynamic value. Strong and moderate correlations were found with MPV and CRP, respectively, and an inverse association with platelet count, but not with PCT. Moreover, IPF levels were higher in culture-positive cases compared to culture-negative ones (13.1% vs. 9.8%; *p* = 0.017) and exhibited diagnostic performance comparable to CRP in predicting blood culture positivity. **Conclusions**: This study presents original and clinically relevant data supporting IPF as a promising and practical hematologic biomarker for early detection and treatment monitoring of neonatal sepsis. Its integration into standard sepsis evaluation protocols may improve early risk stratification and clinical decision-making in neonatal intensive care settings.

## 1. Introduction

Neonatal sepsis is a potentially life-threatening systemic condition characterized by a dysregulated inflammatory response to an invasive microbial infection, occurring within the first 28 days of life [1,2]. Despite advances in neonatal intensive care and antimicrobial therapy, it continues to be a major cause of morbidity and mortality, particularly among preterm and very-low-birth-weight infants [1,2,3].

Timely diagnosis of neonatal sepsis is crucial yet remains challenging due to its nonspecific clinical presentation and significant overlap with non-infectious conditions. Blood cultures, although considered the gold standard, are limited by delayed results, low sensitivity in neonates, and susceptibility to contamination [3,4]. To enhance diagnostic accuracy, a wide range of conventional sepsis biomarkers—such as C-reactive protein (CRP), procalcitonin (PCT), various interleukins, and hematological indices—have been extensively studied. However, none have demonstrated sufficient sensitivity, specificity, or reliability to be used alone in routine clinical practice [5,6].

Due to the limitations of conventional biomarkers, recent studies have explored hematologic parameters derived from automated complete blood count (CBC) analysis. Among these, the immature platelet fraction (IPF) has gained attention as a real-time indicator of thrombopoietic activity. While IPF is widely applied in the clinical assessment of thrombocytopenia, its diagnostic relevance has also been recognized in inflammatory and infectious diseases, including sepsis in adults, where it may offer improved accuracy over CRP and PCT [7,8,9,10,11].

Compared to other platelet indices that have been more extensively studied in neonatal infections [12,13], IPF has received relatively limited research attention, despite reports of elevated levels in septic preterm infants [14,15,16,17,18]. Since thrombocytopenia frequently accompanies sepsis, the ability to detect IPF elevations prior to a decline in platelet count may facilitate earlier diagnosis and improved clinical outcomes [8,9,15]. The present study aims to evaluate the diagnostic utility of IPF as an early marker of infection in neonates with suspected sepsis—specifically before the onset of thrombocytopenia—and to compare its performance with that of conventional sepsis biomarkers.

## 2. Materials and Methods

### 2.1. Study Design and Population

This prospective study was conducted between June 2024 and June 2025 in the Neonatal Intensive Care Unit (NICU) of Recep Tayyip Erdogan University Hospital. The study protocol was approved by the ethics committee of the university. Written informed consent was obtained from the parents or legal guardians of all participating neonates prior to enrollment.

The study population was categorized into three groups: the early-onset sepsis (EOS) group, the late-onset sepsis (LOS) group—collectively referred to as the sepsis group—and a control group. EOS was defined as sepsis occurring within the first 72 h after birth, typically resulting from vertical transmission of pathogens—such as *Group B Streptococcus* or *Escherichia coli*—from the maternal genital tract during labor or delivery. In contrast, LOS was defined as sepsis occurring after 72 h of life and was primarily associated with nosocomial infections. It is often linked to prolonged hospitalization, invasive procedures, or the use of indwelling medical devices in the NICU [1,2,3]. Preterm birth was defined as delivery before 37 completed weeks of gestation (i.e., less than 259 days) from the first day of the woman’s last menstrual period [19].

The control group consisted of neonates hospitalized for non-infectious conditions. Infants with clinical conditions known to affect hematological parameters—such as intrauterine growth restriction, maternal hypertensive disorders, birth asphyxia, meconium aspiration, polycythemia (hematocrit > 65%), intraventricular hemorrhage, pneumothorax, congenital anomalies (including chromosomal anomalies, comorbidities), or hemolytic disease due to Rh or ABO incompatibility—were excluded [1,2,3,20].

### 2.2. Blood Sampling and Analysis

Venous blood samples were collected at the time of sepsis diagnosis and again at 48–72 h after the initiation of antibiotic therapy simultaneously into ethylenediaminetetraacetic acid (EDTA) tubes for CBC and IPF analysis, and into plain serum tubes for CRP and PCT measurements. Blood cultures were obtained using Bactec bottles prior to antibiotic administration. Hematological analyses from the EDTA tubes included standard CBC variables as well as platelet indices, such as mean platelet volume (MPV), plateletcrit, and platelet distribution width (PDW) measured with a multi-parameter automated hematology analyzer. Samples obtained at the time of sepsis and 48–72 h after initiation of antibiotic therapy were designated as sample 1 and sample 2, respectively (e.g., IPF-1 and IPF-2) in the sepsis groups. Samples for the control group were collected between 24 and 48 h after birth and referred to as baseline sample 1. Laboratory tests were conducted on samples collected during routine care, without the need for additional blood sampling.

### 2.3. Sepsis Criteria and Diagnosis

Sepsis was defined as the presence of at least two clinical signs and at least two abnormal laboratory parameters in the context of a proven or suspected infection. Proven sepsis required a positive blood culture, while clinical sepsis was diagnosed in culture-negative cases meeting these criteria [1,2,3,20].

The clinical signs evaluated in this study included respiratory distress (tachypnea, apnea, desaturation, or increased ventilator requirement), cardiovascular instability (bradycardia, pallor, poor perfusion, or hypotension), metabolic abnormalities (hypoglycemia, hyperglycemia, or metabolic acidosis), temperature instability (hypothermia or hyperthermia), feeding intolerance, and neurological symptoms (lethargy, hypotonia, or decreased activity). Supportive laboratory parameters for sepsis, excluding platelet count, were as follows: white blood cell count (WBC) <5.000/mm^3^ or >20.000/mm^3^; absolute neutrophil count (ANC) <1.000/mm^3^ or >17.000/mm^3^; immature-to-total neutrophil ratio >0.2; CRP >15 mg/L; and PCT ≥2 ng/mL [1,2,3,20]. A platelet count below 100,000/mm^3^ was defined as thrombocytopenia and was applied as an exclusion criterion. Additionally, hematologic conditions accompanying sepsis that could confound IPF interpretation—such as polycythemia, hemolysis, thrombosis, and coagulopathy—were also excluded [21].

### 2.4. Measurement of IPF

For IPF measurement, 2–3 mL of venous blood was collected into EDTA tubes and analyzed using the Mindray BC-6000 automated hematology analyzer (Mindray Bio-Medical Electronics Co., Ltd., Shenzhen, China), which utilizes flow cytometry technology. The device employs a fluorescent dye that binds to platelet RNA, allowing the identification of immature platelets based on their larger size and increased fluorescence. Scatter plots are generated to distinguish platelet populations according to cell volume and RNA content. IPF is expressed as the percentage of reticulated (immature) platelets relative to the total platelet count [7].

### 2.5. Statistical Analyses

Statistical analyses were conducted using IBM SPSS Statistics version 29.0 (IBM Corp., Armonk, NY, USA) and MedCalc Statistical Software version 19.1.3 (MedCalc Software bv, Ostend, Belgium; https://www.medcalc.org, accessed on 15 April 2025). The distribution of continuous variables was assessed using the Kolmogorov–Smirnov test. Data were presented as mean ± standard deviation (SD), median (minimum–maximum), or counts and percentages, as appropriate. For between-group comparisons, the Student’s *t*-test or Mann–Whitney U test was applied depending on the distribution of variables. Categorical variables were analyzed using Fisher’s exact test or the Chi-square test. A paired-samples *t*-test and Wilcoxon signed-rank test were used for within-group comparisons over time. The diagnostic performance of parameters was assessed using receiver operating characteristic (ROC) curve analysis. Area under the curve (AUC), sensitivity, specificity, and optimal cut-off values were calculated. The optimal thresholds were determined based on the maximum Youden index (J = Sensitivity + Specificity − 1). Correlations between continuous variables were analyzed using Spearman’s rank correlation coefficient. Logistic regression analysis was performed to identify independent predictors of neonatal sepsis. A *p*-value less than 0.05 was considered statistically significant.

## 3. Results

### 3.1. Demographic and Initial Laboratory Characteristics

**Figure 1:**
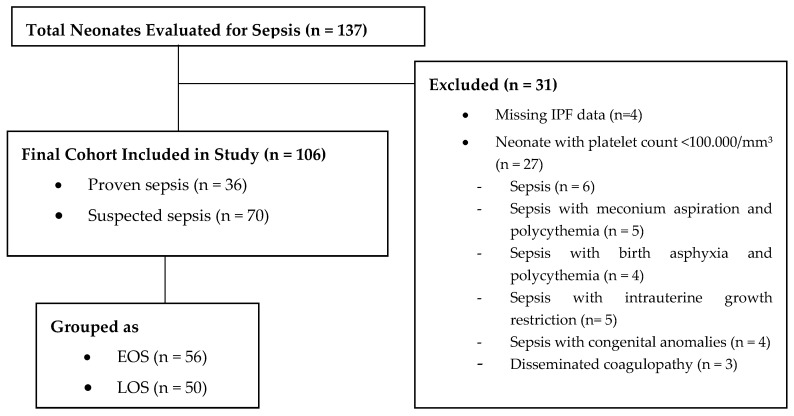
Flowchart of neonatal sepsis enrollment exclusion criteria, and sepsis group classification based on IPF evaluation.

During the study period, 3.261 neonates were admitted to the NICU, of whom 137 (4.2%) were evaluated for suspected sepsis. Of these, 31 were excluded due to missing IPF data (n = 4) or a platelet count <100,000/mm^3^ (n = 27), including cases with isolated sepsis or sepsis with comorbidities. The final cohort comprised 106 neonates with sepsis (36 proven, 70 suspected), categorized as 56 in the EOS group and 50 in the LOS group, and was compared with a control group of 44 neonates (Figure 1).

Gestational age and birth weight were significantly lower in the LOS group compared to both the EOS and the control groups (*p* < 0.001), indicating a higher rate of preterm infants in the LOS group. There were no significant differences among the groups in terms of sex or mode of delivery.

Regarding the time of sepsis laboratory parameters, CRP-1, PCT-1, and IPF-1 levels were significantly higher in the EOS and LOS groups compared to controls. Median IPF-1 values were 9.2% in the EOS group and 14.6% in the LOS group, while the control group had a median of 3.7% (*p* < 0.001). In a similar pattern, MPV-1 was significantly higher in both sepsis groups, while ANC-1 was reduced in the LOS group compared to the EOS and control groups (*p* < 0.001 and *p* = 0.004, respectively). Blood culture positivity was markedly higher in the LOS group (70%) than in the EOS group (1.8%) (*p* < 0.001) (Table 1).

Among preterm infants in our cohort, subgroup analysis revealed that the median gestational age remained lower in the LOS group [27.6 weeks (23–35.2)] compared to the EOS [32.2 weeks (25–36.4)] and control groups [34.4 weeks (31–36.5)] (*p* = 0.028). The median IPF was significantly higher in the LOS group [18.8% (14.1–32.3%)] compared to the EOS [14.5% (12.8–39.2%)] and control groups [4.2% (1.3–6.5%)] (<0.001) (not included in the table).

Hematological indices also demonstrated group-specific differences. Platelet counts were significantly lower in both sepsis groups compared to controls, particularly in LOS neonates (*p* = 0.016), while plateletcrit-1 and PDW-1 levels showed modest but significant alterations across groups (*p* = 0.008 and *p* = 0.009, respectively). MCV-1 was elevated in EOS infants (*p* < 0.001), and although WBC-1 levels did not differ significantly, trends were consistent with inflammatory activation (Table 1).

### 3.2. Temporal Changes in Inflammatory and Hematological Parameters

Temporal evaluation revealed that CRP, PCT, and IPF levels significantly decreased from the time of diagnosis to 48–72 h after antibiotic initiation in both EOS and LOS groups, with higher initial values observed in LOS neonates (*p* < 0.001 for all). ANC levels also declined significantly over time in both groups. Platelet counts showed a modest increase, which reached statistical significance only in the LOS group. MPV and MCV values exhibited significant temporal changes, whereas PDW and plateletcrit remained largely unchanged (Table 2).

### 3.3. Diagnostic Performance of IPF in Comparison with CRP and PCT Based on ROC Analysis

**Figure 2:**
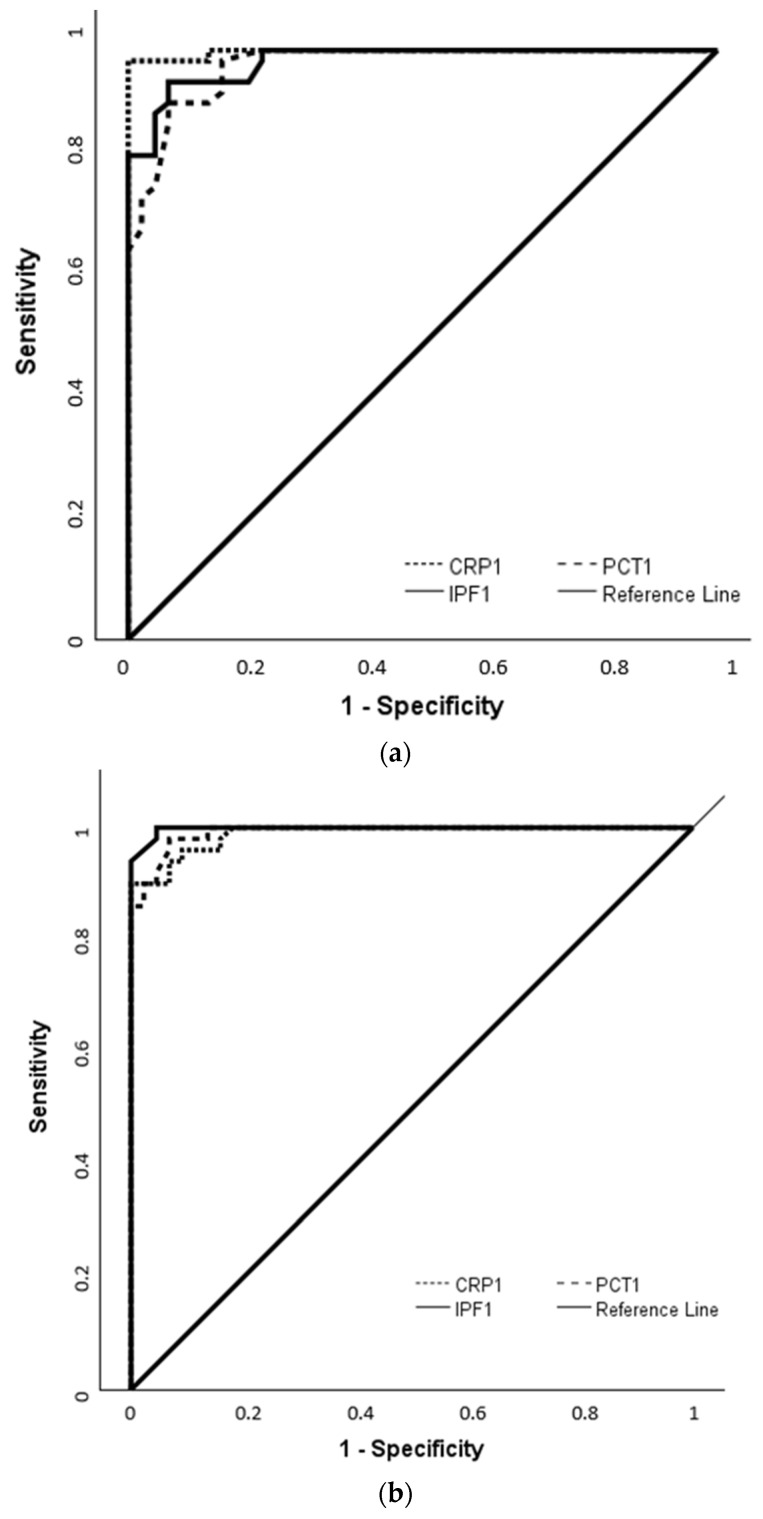
Diagnostic performance of IPF in comparison with CRP and PCT based on ROC analysis in EOS and LOS groups of this study; (**a**) ROC curves for EOS group; (**b**) ROC curves for LOS group.

To evaluate the diagnostic performance of IPF in comparison with CRP and PCT, ROC analysis was conducted using measurements obtained at the time of sepsis diagnosis in both EOS and LOS groups. In the EOS group, IPF-1 showed an AUC of 0.982, with a cut-off value of 5.5% yielding 94.6% sensitivity and 93.2% specificity (*p* < 0.001). In the LOS group, IPF-1 had an AUC of 0.998, and at a cut-off value of 6%; it achieved 100% sensitivity and 95.5% specificity (*p* < 0.001). For CRP-1 and PCT-1, cut-off values of 14 mg/L and 1.9 ng/mL in EOS and 13.75 mg/L and 1.9 ng/mL in LOS provided sensitivities and specificities above 90%, with AUCs ranging from 0.975 to 0.998 (Figure 2a,b, Table 3).

### 3.4. Correlation Analysis of IPF with Hematological and Inflammatory Marker

Correlation analysis was performed to examine the associations between IPF and various hematological and inflammatory markers across the study groups. In the EOS group, IPF was moderately to strongly correlated with WBC (r =0.428), ANC (r = 0.360), and MPV (r = 0.567) (*p* < 0.01 for all), and weakly inversely correlated with platelet count (r = –0.248, *p* = 0.065). In the LOS group, IPF showed weaker but significant correlations with MPV (r = 0.286, *p* = 0.044) and platelet count (r = –0.284, *p* = 0.046), but no association with inflammatory markers. When both groups were analyzed together as the sepsis group (*n* = 106), IPF demonstrated significant positive correlations with CRP (r = 0.396, *p* < 0.001), MPV (r = 0.605, *p* < 0.001), PDW (r = 0.265, *p* = 0.001), and WBC (r = 0.192, *p* = 0.019), and a negative correlation with platelet count (r = –0.266, *p* = 0.001). No significant correlations were observed between IPF and PCT, ANC, plateletcrit, or MCV (*p* > 0.05 for all). 

### 3.5. Logistic Regression of Sepsis Predictors

In the sepsis cohort (*n* = 106), logistic regression analysis revealed several clinical and laboratory variables as independent predictors of neonatal sepsis. IPF was a strong predictor (OR = 6.675, *p* < 0.001), along with PCT (OR = 25.088, *p* < 0.001), CRP (OR = 2.425, *p* < 0.001), MPV (OR = 2.622, *p* < 0.001), and PDW (OR = 3.576, *p* = 0.005). Platelet count showed a statistically significant association (*p* = 0.035), though the effect size was negligible (OR = 1.000). Among clinical variables, preterm birth was significantly associated with sepsis (OR = 2.118, *p* = 0.041) (Table 4).

### 3.6. Comparison of Culture-Positive and Culture-Negative Neonates

Among the sepsis cohort (*n* = 106), 36 neonates (34%) had positive blood cultures. Compared to culture-negative cases, neonates with positive blood cultures had significantly lower birth weights; gestational ages; WBC, ANC, and MCV values; as well as significantly higher MPV, CRP, and IPF-1 levels [13.1% (6.1–32.3) vs. 9.8% (4.4–39.2); *p* = 0.017]. Platelet count and plateletcrit, PDW, and PCT levels did not differ significantly between the groups (Table 5).

In this study, the most frequently isolated pathogens were coagulase-negative staphylococci (CoNS), including both methicillin-sensitive and methicillin-resistant strains, identified in 18 cases (16.4%), followed by *Klebsiella pneumoniae* (*n* = 10, 9.1%), *Escherichia coli* (*n* = 3, 2.7%), *Staphylococcus aureus* (*n* = 2, 1.8%), *Candida glabrata, Pseudomonas aeruginosa*, and Enterobacter species (*n* = 1 each, 0.9%). With the exception of a single *Escherichia coli* isolate in the EOS group, all culture-positive cases were confined to the LOS group.

### 3.7. ROC-Based Assessment of IPF and CRP for Identifying Culture-Positive Cases

**Figure 3:**
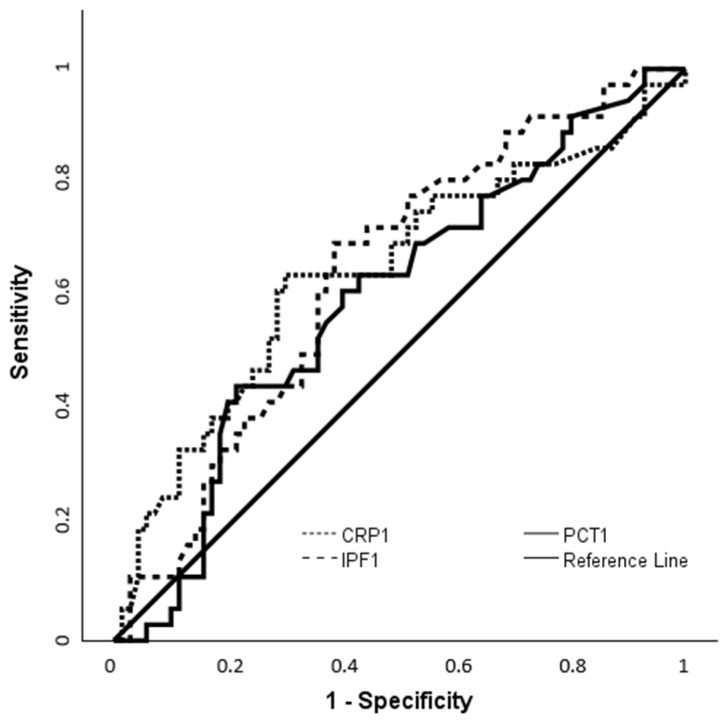
ROC Curve of IPF and CRP for the prediction of blood culture positivity.

To further evaluate the diagnostic performance of initial inflammatory markers in predicting culture positivity, ROC analyses were performed. IPF-1 levels were higher in the culture-positive group (13.1%, 6.1–32.3) than in the culture-negative group (9.8%, 4.4–39.2) (*p* = 0.017) (Table 5). At a cut-off value of 10.85%, IPF-1 yielded 69.4% sensitivity and 61.4% specificity (*p* = 0.010). CRP-1 was also elevated in culture-positive neonates compared to the culture-negative group, with 63.9% sensitivity and 70% specificity at a cut-off of 29.9 mg/L. PCT-1 levels, however, did not show a significant difference between groups (*p* = 0.100) (Figure 3, Table 6).

## 4. Discussion

This study evaluated the value of IPF as an early hematologic indicator in neonatal sepsis and demonstrated its strong diagnostic performance. IPF levels were significantly higher in both the EOS and LOS groups compared to controls. ROC analysis showed favorable sensitivity and specificity in detecting sepsis and predicting blood culture positivity. Moreover, logistic regression confirmed IPF as an independent risk factor, performing comparably or even superior to conventional markers such as CRP and PCT. IPF also exhibited significant positive correlations with established inflammatory and platelet-related parameters, including CRP, WBC, and MPV. Given that the literature on IPF in neonatal sepsis is still emerging, our findings provide compelling preliminary evidence supporting its utility as a sensitive and readily accessible early biomarker that may precede the onset of thrombocytopenia.

Defined as a marker of early thrombopoiesis, IPF represents the proportion of newly released, reticulated platelets containing residual RNA and rough endoplasmic reticulum. Advances in automated hematology analyzers have facilitated its routine measurement. While initially studied in adults for thrombopoietic activity, cardiovascular risk, and early sepsis detection, IPF is increasingly used in pediatrics to monitor bone marrow recovery, assess thrombocytopenia, and differentiate between hypo- and hyperproliferative platelet disorders [7,8,9,10,11,22,23,24,25,26]. Its potential as an early biomarker for neonatal sepsis is gaining interest, though current evidence remains limited [14,15,16,17,18].

While IPF is increasingly recognized as a promising early biomarker for neonatal sepsis, its interpretation must consider physiological factors affecting neonatal reference values, such as gestational age, sampling method, and clinical status [7,9,16]. Garofoli et al. [16] reported higher IPF levels in preterm than term neonates, with inverse correlations to gestational age and platelet count [16]. Other studies have reported mean values ranging from 1.5% to 4.1%, depending on sampling timing and methodology [14,27,28]. In our study, the control group consisted of 25 preterm and 19 term neonates, with an overall median IPF value of 3.7% (range: 0.9–6.5). Subgroup analysis demonstrated slightly higher IPF levels in preterm infants, with a median of 4.2% (range: 1.3–6.5), compared to 3.1% (range: 0.9–5.2) in term infants. Despite the limited sample size, these findings indicate gestational age–dependent variability in baseline IPF, aligning with previously cited physiological reference ranges. Considering these data, the elevated IPF observed among preterm infants with sepsis appears to be largely driven by the infectious process. However, the lower gestational age in the LOS group compared to the control group (median GA: 27.6 vs. 34.4 weeks, *p* = 0.028) may have also contributed to this difference.

The link between elevated IPF levels and neonatal sepsis has been attributed to specific immunohematologic pathways, underscoring its potential as an early and sensitive indicator of infection-induced platelet production. In the early phase of sepsis, innate immune responses stimulate platelet activation through toll-like receptors, leading to interleukin-1β (IL-1β) release and the formation of neutrophil extracellular traps that help contain pathogens [29]. Concurrently, elevated thrombopoietin levels enhance megakaryopoiesis, resulting in the accelerated production and release of immature platelets into circulation. Together, these inflammatory and hematopoietic processes contribute to a rise in IPF that often precedes clinically evident thrombocytopenia, pointing to its diagnostic value in the early identification of sepsis [30].

The importance of IPF as a marker of infection-induced thrombopoietic response was initially established in adult populations. Briggs et al. published reference IPF% ranges in healthy individuals (mean: 3.4%; range: 1.1–6.1%) [22]. Subsequent studies by De Blasi et al. [8] and Di Mario et al. [29] proposed that IPF could serve as an early indicator, detecting patients before clinical signs of sepsis become evident. Enz Hubert et al. documented significantly elevated IPF levels in adults with severe sepsis (6.2 ± 3.0%) compared to milder infections (3.6 ± 2.6%) [9], while Park et al. confirmed higher IPF values in septic patients relative to healthy controls (4.9% vs. 2.9%, *p* < 0.001) [11].

A similar trend was observed in our neonatal cohort, with significantly elevated IPF levels in both EOS and LOS groups compared to controls [median: 9.2% in EOS, 14.6% in LOS, and 3.7% in controls; *p* < 0.001]. Likewise, Septiane et al. reported increased IPF in EOS neonates relative to controls (5.6% vs. 3.79%; *p* < 0.001) at 48–72 h of age, although the lack of detailed platelet count data may have constrained the assessment of thrombopoietic dynamics [17]. Indra et al. noted higher IPF levels in LOS than EOS (10.9% vs. 7.7%; *p* = 0.001), attributing the discrepancy to increased inflammatory burden [18]. Consistently, our data also demonstrated higher IPF in LOS, potentially reflecting intensified and sustained inflammatory response in late-onset cases. These findings support the role of IPF as a reliable early-phase biomarker in neonatal sepsis and enhance its diagnostic utility in differentiating sepsis subtypes.

The increase in IPF observed in our study preceded the onset of thrombocytopenia and revealed excellent diagnostic performance, with AUCs of 0.98 in EOS (cut-off: 5.5%, 95% sensitivity, 93% specificity) and 0.99 in LOS (cut-off: 6.0%, 100% sensitivity, 96% specificity). The abovementioned study by Septiane et al., delta IPF—defined as the difference in IPF between the first ≤6 h and 48–72 h of life—was evaluated as a predictive marker for EOS. A threshold >0.4% yielded 64.4% sensitivity, 70.0% specificity, and a 2.39-fold increased risk of EOS [17]. Our findings similarly demonstrated the dynamic nature of IPF in response to treatment. A significant decline in IPF levels—reflected by a notable delta IPF—was observed 48–72 h after the initiation of antibiotic therapy (*p* < 0.001). Although our primary analysis focused on absolute IPF values at diagnosis, the observed delta IPF highlights its potential as a dynamic marker of treatment response and suggests the integration of both static and temporal measures in the evaluation of neonatal sepsis.

In line with these data, Liu et al. [10] reported a diagnostic threshold of 5.5%—identical to the optimal cut-off in our study—but with lower diagnostic performance (84.2% sensitivity and 72.2% specificity) in an adult population. They further found that higher IPF levels were associated with infection severity, and that their combination with CRP or PCT enhanced early diagnostic performance [10]. In parallel, we observed elevated CRP and PCT levels alongside IPF in our cohort, with cut-off values consistent with those reported in the literature [20]. These findings reinforce the potential role of IPF in multi-marker diagnostic approaches, especially for the early detection of neonatal sepsis before the onset of thrombocytopenia.

However, in both the EOS and LOS subgroups of our neonatal cohort, IPF did not demonstrate the expected correlation with CRP or PCT. In EOS, it correlated positively with WBC, ANC, and MPV, and inversely with platelet count; in LOS, only MPV and platelet count were associated. In the overall sepsis cohort, IPF correlated strongly with MPV, moderately with CRP, and inversely with platelet count, but not with PCT, ANC, plateletcrit, or MCV. These patterns indicate that IPF primarily reflects hematopoietic activation rather than conventional inflammatory markers. Its strong correlation with MPV—a known indicator of bone marrow activity—may reflect compensatory thrombopoiesis in response to peripheral platelet consumption in sepsis. This is supported by prior evidence from Nam et al., who demonstrated that both IPF and MPV outperformed CRP in predicting pediatric sepsis [31]. Additional evidence comes from Mishra et al., who found higher maternal IPF and MPV levels in pregnancies complicated by neonatal sepsis and respiratory distress, although conventional inflammatory markers were not assessed (IPF: 10.11 ± 6.27% vs. 5.06 ± 4.07% in sepsis vs. non-sepsis; 9.11 ± 6.38% vs. 5.54 ± 4.43% in respiratory distress vs. non-distress) [32].

Moreover, in our study, IPF demonstrated diagnostic performance comparable to traditional inflammatory markers such as CRP and PCT, with the added advantage of earlier elevation. Among all variables included in the logistic regression model, IPF exhibited one of the strongest independent associations with sepsis (OR = 6.675; 95% CI: 2.925–15.234; *p* < 0.001). PCT had the highest predictive value (OR = 25.088), and CRP remained statistically significant (OR = 2.425), pointing to a rapid hematologic response to infection and supporting the clinical utility of IPF due to its availability through routine blood counts and its sensitivity to early bone marrow activation.

Elucidating the distinct biological mechanisms underlying these biomarkers may help explain their divergent correlations and complementary roles in sepsis diagnosis. Although IPF and CRP originate from distinct biological pathways, both increase during early inflammation—CRP as a hepatic acute-phase reactant induced by IL-6, and IPF as a hematologic response to inflammation-driven thrombopoietin release [2,5,30]. This may account for the moderate correlation observed between them in our sepsis cohort, suggesting that IPF reflects both inflammatory and hematopoietic activity. In contrast, PCT is induced by bacterial endotoxins and proinflammatory cytokines (e.g., IL-1β, TNF-α) and does not directly relate to thrombopoiesis [2,5]. The lack of correlation between IPF and PCT likely shows their differing biological roles and response kinetics.

An important additional observation in our study was that preterm birth itself emerged as an independent risk factor for neonatal sepsis (OR = 2.118, *p* = 0.041), alongside IPF. In the preterm subgroup, the markedly elevated IPF levels observed in the LOS group initially raised the question of whether lower gestational age might have confounded this association. While prematurity may have contributed to increased IPF levels through enhanced baseline thrombopoietic activity, the strength of the association between IPF and sepsis was substantially greater. This underscores the necessity of interpreting IPF values within the context of gestational age—particularly in non-infected preterm neonates—to avoid misattribution of physiological elevations to infectious processes.

Another finding of the study was that CoNS were the most frequently isolated pathogens, followed by *Klebsiella pneumoniae, Escherichia coli*, and other Gram-negative or fungal organisms. This distribution aligns with previous reports identifying CoNS as the primary cause of LOS, particularly in preterm infants with central lines [1,2,3]. Gram-positive bacteria —primarily CoNS and Staphylococcus aureus— accounted for 55.6% of culture-positive cases. To further evaluate the clinical applicability of IPF, we analyzed its association with blood culture positivity—the gold standard for confirming neonatal sepsis—and found significantly higher IPF levels in culture-positive neonates compared to culture-negative cases (13.1% vs. 9.8%; *p* = 0.017), predominantly in LOS cases.

Consistent with these data, Di Mario et al. reported elevated IPF levels in culture-positive adult sepsis cases, 84% of which were due to Gram-positive organisms (4.86% vs. 1.79%) [29]. The similarity in IPF response patterns across age groups may reflect a recurring association between Gram-positive bloodstream infections and early thrombopoietic activation, suggesting that IPF could represent a universally applicable and early indicator of bacterial sepsis.

Additionally, IPF and CRP demonstrated comparable diagnostic performance in predicting blood culture positivity. An IPF cut-off of 10.85% yielded 69.4% sensitivity and 61.4% specificity (AUC = 0.642), while CRP at 29.9 mg/L achieved 63.9% sensitivity and 70% specificity (AUC = 0.645). These CRP results are broadly consistent with previous reports, where sensitivities ranged from 60% to 90%, and specificity varied depending on timing and clinical context [33,34,35,36]. In CoNS bacteremia, especially in very preterm infants, attenuated inflammatory responses may lower CRP levels; however, this may increase the risk of false negatives [1,2,3,4,5,36]. PCT, by contrast, was not significantly associated with culture positivity (AUC = 0.589, *p* = 0.100), likely reflecting its physiological elevation during the first 48 h of life—even in the absence of infection—which limits its specificity in EOS [3,4,5,37]. Such limitations underscore the value of complementary biomarkers like IPF, which appears less affected by early postnatal physiological changes and may serve as a more stable and reliable marker for the early identification of neonatal sepsis.

Studies in both adult and neonatal populations have shown that PCT levels are typically higher in Gram-negative bacteremia, largely due to lipopolysaccharide (LPS)-induced inflammatory responses. LPS stimulates PCT synthesis in various tissues, including the lungs, liver, and intestines. In contrast, Gram-positive cocci primarily release exotoxins that are protein-based and structurally less stable, often eliciting a milder PCT response. These pathogen-specific differences may explain the lower PCT levels observed in Gram-positive infections, although the underlying molecular mechanisms remain incompletely understood [37,38,39].

### Strengths and Limitations

To the best of our knowledge, this study provides novel insights into the diagnostic value of IPF in neonatal sepsis. Nonetheless, several limitations merit consideration. As a single-center study, generalizability may be limited. The small number of culture-positive EOS cases also reduced the robustness of subgroup analyses, likely due to intrapartum maternal antibiotic administration, which is known to lower blood culture yields [40]. Additionally, neonates with severe thrombocytopenia were excluded to avoid confounding IPF interpretation; although this may have introduced selection bias, it enabled a more accurate evaluation of IPF dynamics during the early phase of infection. While IPF was measured both at the time of sepsis diagnosis (i.e., initiation of antibiotic therapy) and 48–72 h later, limited follow-up—due to clinical and ethical constraints—precluded a detailed assessment of its clearance kinetics during infection resolution. Another methodological consideration is the gestational age difference between the LOS and control groups, which—partly due to sample size constraints—may have subtly influenced IPF levels through prematurity-related effects. However, both IPF and preterm birth emerged as independent predictors of sepsis in our analysis; the association was considerably stronger for IPF, underscoring the need to interpret IPF values in the context of gestational age—particularly in preterm neonates without clinical evidence of infection. Larger, multicenter studies with extended follow-up are warranted to validate these findings and further explore the prognostic utility of IPF.

## 5. Conclusions

Our findings highlight the clinical utility of IPF as a practical and informative biomarker for the early detection and management of neonatal sepsis. Its significant elevation in both early- and late-onset sepsis, independent predictive value, and measurable decline following antimicrobial therapy suggest that IPF may serve not only as a diagnostic indicator but also as a dynamic marker for monitoring treatment response. Moreover, its higher levels in culture-positive cases and diagnostic performance comparable to CRP reinforce its potential as a surrogate marker while awaiting microbiological confirmation. Early responsiveness to marrow activation makes it especially valuable in the initial phase of infection, possibly before CRP or PCT elevation.

Given its routine availability via complete blood count analysis and diagnostic performance approaching that of CRP and PCT, IPF may enhance early risk assessment and guide timely clinical decisions in NICU settings. However, it should complement existing markers until further validated.

## Figures and Tables

**Table 1:** Demographic and initial laboratory characteristics of study groups.

Characteristic	Control Group *n* = 44 ^1^	EOS Group *n* = 56 ^1^	LOS Group *n* = 50 ^1^	*p*
Gestational weeks	35.5 (31–41)	37.1 (25–41.5)	30 (23.3–40.3)	<0.001 ^b,c,^*
Preterm birth	25 (56.8)	27 (48.2)	45 (90)	<0.001 ^b,c,^*
Birth weight (kg)	2.55 (1.32–4.08)	2.73 (0.73–4.10)	1.32 (0.54–3.9)	<0.001 ^b,c,^*
Male	32 (72.7)	34 (60.7)	27 (54)	0.17 *
Cesarean delivery	34 (77.3)	38 (67.9)	40 (80)	0.32 *
WBC-1 (×10^3^/µL)	12.07 (4.77–25.65)	13.91 (4.06–37.14)	11.82 (1.58–47.14)	0.358 *
ANC-1 (×10^3^/µL)	5.41 (1.55–15.75)	8.39 (0.65–35.31)	4.30 (1.03–30.36)	0.004 ^b,^*
Platelet-1 (×10^3^/µL)	287.5 (130–644)	218 (118–677)	221 (110–509)	0.016 ^a,^*
MCV-1 (fL)	100.6 ± 7.2	102.8 ± 6.7	96.9 ± 9.1	**<0.001 ^b,&^**
MPV-1 (fL)	10.6 ± 1	11.3 ± 1.2	12.9 ± 1.2	**<0.001 ^a,b,c,&^**
Plateletcrit-1 (%)	0.31 (0.09–0.73)	0.24 (0.10–0.78)	0.32 (0.02–1.15)	0.008 ^a,b,^*
PDW-1 (%)	16.5 ± 0.4	16.8 ± 0.5	16.7 ± 0.4	**0.009 ^a,&^**
IPF-1 (%)	3.7 (0.9–6.5)	9.2 (4.4–39.2)	14.6 (6.1–32.3)	<0.001 ^a,b,c,^*
CRP-1 (mg/L)	6.5 (0.3–13.5)	25.1 (11.2–100)	35.8 (10–150)	<0.001 ^a,c,^*
PCT-1 (ng/mL)	0.75 (0.08–2.6)	4.1 (1.2–100)	5.4 (1.6–50)	**<0.001 ^a,c,^**
Positive blood culture	0 (0)	1 (1.8)	35 (70)	<0.001 ^b,c,^*

WBC: white blood cell; ANC: absolute neutrophil count; MCV: mean corpuscular volume; MPV: mean platelet volume; PDW: platelet distribution width; IPF: immature platelet fraction; CRP: C-reactive protein; PCT: procalcitonin; ^1^: number (percentage), mean ± SD, median (min–max); sample 1: sample obtained at the time of sepsis (for sepsis groups) or baseline sample (for control group); *: Mann–Whitney U test, ^&^: Student’s *t*-test; ^a^: EOS group vs. control group; ^b^: EOS group vs. LOS group; ^c^: LOS group vs. control group.

**Table 2:** Temporal changes in inflammatory and hematologic markers in EOS and LOS groups.

Markers	EOS Group *n* = 56 ^1^	LOS Group *n* = 50 ^1^	*p*
WBC-1 (×10^3^/µL)	13.91 (4.06–37.14)	11.82 (1.58–47. 14)	0.414 *
WBC-2 (×10^3^/µL)	10.44 (1.67–27.19)	10.28 (4.24–38.00)	
***p *** **^¥^**	**0.010**	0.233	
ANC-1 (×10^3^/µL)	8.39 (0.65–35.31)	4.30 (1.03–30.36)	0.080 *
ANC-2 (×10^3^/µL)	4.53 (0.60–22.11)	3.25 (1.27–16.17)	
***p *** **^¥^**	**<0.001**	**0.012**	
Platelet-1 (×10^3^/µL)	218 (118–677)	221 (115–509)	0.535 *
Platelet -2 (×10^3^/µL)	252.5 (211–320)	288 (110–656)	
***p *** **^¥^**	0.088	**0.032**	
MCV-1 (fL)	102.8 ± 6.7	96.9 ± 9.1	**0.016** ^&^
MCV-2 (fL)	101.7 ± 6.3	93.7 ± 8.2	
***p *** **^§^**	**0.038**	**<0.001**	
MPV-1 (fL)	11.3 ± 1.2	12.9 ± 1.2	**0.006** ^&^
MPV-2 (fL)	11.6 ± 1.3	12.5 ± 1.2	
***p *** **^§^**	**0.040**	0.069	
Plateletcrit-1 (%)	0.24 (0.10–0.78)	0.32 (0.02–1.16)	0.571 *
Plateletcrit-2 (%)	0.31 (0.10–0.82)	0.36 (0.12–0.93)	
***p *** **^¥^**	**0.006**	0.203	
PDW-1 (%)	16.7 (16–18.2)	16.8 (15.7–17.4)	0.959 *
PDW-2 (%)	16.7 (15.9–18.1)	16.6 (15.9–17.6)	
***p *** **^¥^**	0.677	0.781	
IPF-1 (%)	9.2 (4.4–39.2)	14.6 (6.1–32.3)	0.013 *
IPF-2 (%)	6.4 (1.4–32.3)	8.95 (3–23.7)	
***p *** **^¥^**	**<0.001**	**<0.001**	
CRP-1 (mg/L)	25.1 (11.2–100)	35.8 (10–150)	0.058 *
CRP-2 (mg/L)	6.02 (1.3–45.7)	8 (1.3–58)	
***p *** **^¥^**	**<0.001**	**<0.001**	
PCT-1 (ng/mL)	4.1 (1.2–100)	5.4 (1.6–50)	0.173 *
PCT-2 (ng/mL)	1.1 (0.1–16)	1.4 (0.1–14)	
***p *** **^¥^**	**<0.001**	**<0.001**	

WBC: white blood cell; ANC: absolute neutrophil count; MCV: mean corpuscular volume; MPV: mean platelet volume; PDW: platelet distribution width; IPF: immature platelet fraction; CRP: C-reactive protein; PCT: procalcitonin; ^1^ mean ± SD, median (min–max); sample 1: sample obtained at the time of sepsis; sample 2: sample obtained 48–72 h after initiation of antibiotic therapy. *p* */&: the differences of between-group Δ values analyzed with Mann–Whitney U test * or Student’s *t*-test &; *p* §/¥: the differences of in-group Δ values analyzed with paired-samples *t*-test § or Wilcoxon signed rank test ¥.

**Table 3:** Diagnostic performance of IPF in comparison with CRP and PCT based on ROC analysis in EOS and LOS groups of this study.

Group	Cut-Off	AUC (95% CI)	*p*-Value	Sensitivity (%)	Specificity (%)
**EOS group**					
IPF-1	5.5	0.982 (0.963–1.000)	**<0.001**	94.6	93.2
CRP-1	14	0.998 (0.992–1.003)	**<0.001**	98.2	100
PCT-1	1.9	0.975 (0.951–0.999)	**<0.001**	91.1	93.2
**LOS group**					
IPF-1	6	0.998 (0.994–1.002)	**<0.001**	100	95.5
CRP-1	13.75	0.989 (0.975–1.002)	**<0.001**	90	100
PCT-1	1.9	0.992 (0.981–1.003)	**<0.001**	98	93.2

IPF: immature platelet fraction; CRP: C-reactive protein; PCT: procalcitonin; AUC, area under curve; CI; Confidence Interval; sample 1: sample obtained at the time of sepsis.

**Table 4:** Logistic regression analysis of variables associated with neonatal sepsis in the sepsis cohort.

Variables	OR	95% CI	*p*-Value
Preterm birth	2.118	1.033–4.342	**0.041**
Male Gender	0.508	0.236–1.095	0.084
Cesarean delivery	0.819	0.358–0.819	0.637
WBC-1	1.000	1.000–1.000	0.226
ANC-1	1.000	1.000–1.000	0.247
Platelet-1	1.000	1.000–1.000	**0.035**
MCV-1	0.991	0.948–1.036	0.690
MPV-1	2.622	1.769–3.885	**<0.001**
Plateletcrit-1	0.666	0.075–5.930	0.715
PDW-1	3.576	1.467–8.717	**0.005**
IPF-1	6.675	2.925–15.234	**<0.001**
CRP-1	2.425	1.575–3.732	**<0.001**
PCT-1	25.088	6.970–90.307	**<0.001**

OR: odds ratio; CI: Confidence Interval; WBC: white blood cell; ANC: absolute neutrophil count; MCV: mean corpuscular volume; MPV: mean platelet volume; PDW: platelet distribution width; IPF: immature platelet fraction; CRP: C-reactive protein; PCT: procalcitonin. Sample 1: sample obtained at the time of sepsis.

**Table 5:** Comparison of clinical and laboratory parameters between culture-positive and culture-negative neonates.

Parameters	Culture-Negative Neonates *n* = 70 ^1^	Culture-Positive Neonates *n* = 36 ^1^	*p*-Value
Gestational weeks	35.1 ± 4.9	29.8 ± 3.8	**<0.001 ^&^**
Birth weight (kg)	2453 ± 1025.3	1408.3 ± 589.3	**<0.001 ^&^**
WBC-1 (×10^3^/µL)	13.76 (4.06–37.14)	11.42 (1.58–22.96)	0.025 *
ANC-1 (×10^3^/µL)	7.65 (0.65–35.31)	3.84 (1.03–17.98)	<0.001 *
Platelet-1 (×10^3^/µL)	220 (115- 577)	216.5 (110–488)	0.247 *
MCV-1 (fL)	101.6 (77–124.8)	97.8 (81.5–122)	0.024 *
MPV-1 (fL)	11.5 ± 1.3	13 ± 1.3	**<0.001** ^&^
Plateletcrit-1 (%)	0.3 (0–1.2)	0.3 (0–0.5)	0.610 *
PDW-1 (%)	16.7 ± 0.4	16.7 ± 0.4	0.725 ^&^
IPF-1 (%)	9.8 (4.4–39.2)	13.1 (6.1–32.3)	0.017 *
CRP-1 (mg/L)	25 (10.2–150)	38.3 (10–148)	0.015 *
PCT-1 (ng/mL)	4.1 (1.2–100)	5.4 (1.6–50)	0.100 *

WBC: white blood cell; ANC: absolute neutrophil count; MCV: mean corpuscular volume; MPV: mean platelet volume; PDW: platelet distribution width; IPF: immature platelet fraction; CRP: C-reactive protein; PCT: procalcitonin; sample 1: sample obtained at the time of sepsis; ^1^ mean ± SD, median (min–max); *: Mann–Whitney U test; &: Student’s *t*-test.

**Table 6:** ROC analysis of IPF and CRP for the prediction of blood culture positivity at the time of sepsis diagnosis.

Markers	Cut-Off	AUC (95% CI)	*p*-Value	Sensitivity (%)	Specificity (%)
IPF-1	10.85	0.642 (0.534–0.749)	0.010	69.4	61.4
CRP-1	29.9	0.645 (0.528–0.761)	0.015	63.9	70

IPF: immature platelet fraction; CRP: C-reactive protein; AUC, area under curve; CI, Confidence Interval; sample 1: sample obtained at the time of sepsis.

## Data Availability

The research data are not publicly available.
